# Idiopathic pulmonary fibrosis in BRIC countries: the cases of Brazil, Russia, India, and China

**DOI:** 10.1186/s12916-015-0495-0

**Published:** 2015-09-24

**Authors:** Luca Richeldi, Adalberto Sperb Rubin, Sergey Avdeev, Zarir F. Udwadia, Zuo Jun Xu

**Affiliations:** Southampton Respiratory Biomedical Research Unit, Mailpoint 813, LE75 E Level, South Academic Block, University Hospital Southampton NHS Foundation Trust, Southampton, SO16 6YD UK; Federal University of Porto Alegre (UFCSPA), Pulmonary Department of Santa Casa Hospital, Porto Alegre, Brazil; Clinical Department, Pulmonology Research Institute, 32, 11-th Parkovaya str., Moscow, 105077 Russia; Hinduja Hospital and Research Centre, Mahim, Mumbai 400016 India; Breach Candy Hospital, B. Desai Road, Mumbai, 400026 India; Department of Respiratory Medicine, Peking Union Medical College Hospital, Chinese Academy of Medical Sciences & Peking Union Medical College, Beijing, 100730 China

**Keywords:** BRIC countries, High-resolution computed tomography, Idiopathic pulmonary fibrosis, Nintedanib, Pirfenidone

## Abstract

Idiopathic pulmonary fibrosis (IPF), the prototype of interstitial lung diseases, has the worst prognosis and is the only interstitial lung disease for which approved pharmacological treatments are available. Despite being considered a rare disease, IPF patients pose major challenges to both physicians and healthcare systems. It is estimated that a large number of IPF patients reside in BRIC countries (Brazil, Russia, India, and China) given their overall total population of approximately 3 billion inhabitants. Nevertheless, the limited availability of chest imaging in BRIC countries is considered a chief obstacle to diagnosis, since high-resolution computed tomography of the chest is the key diagnostic test for IPF. Further, obtaining reliable lung function tests and providing treatment access is difficult in the more rural areas of these countries. However, IPF might represent an opportunity for BRIC countries: the exponentially increasing demand for the enrollment of IPF patients in clinical trials of new drugs is predicted to face a shortage of patients – BRIC countries may thus play a crucial role in advancing towards a cure for IPF.

## Introduction

**Fig. 1:**
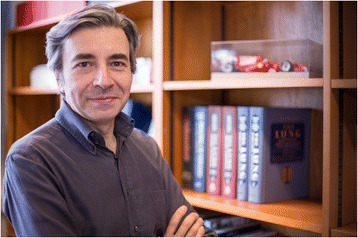
Luca Richeldi is Professor of Respiratory Medicine and Chair of Interstitial Lung Disease at the University of Southampton, UK. He is also a member of the editorial board of *BMC Medicine* and guest editor for the article collection Idiopathic Pulmonary Fibrosis: Diagnosis, Management, and New Therapies

Luca Richeldi (Fig. 1)

Idiopathic pulmonary fibrosis (IPF) is the prototype of interstitial lung diseases (ILDs), a group of pulmonary diseases also referred to as “rare lung diseases”. A rare disease is defined by the European Union as one that affects less than 5 in 10,000 of the general population; as a consequence, a single rare disease may affect only a handful of patients, whereas another may affect as many as 250,000. Therefore, the concept of “rare” needs to be further defined in order to more accurately address diseases and their interventions. Diseases (both common and rare) do not present boundaries or geographic preferences; this is particularly true for diseases, such as IPF, for which risk factors linked to a particular racial background or a specific, defined geographic area or environment have not been identified to date. Thus, it is likely that the burden of disease will be concentrated in the most densely populated regions of the globe. In this context, BRIC countries (Brazil, Russia, India, and China), with an estimated 2.9 billion inhabitants overall, may comprise 1 million cases of a rare disease, thus easily representing a major medical need.

Defining the exact epidemiology of a rare disease is a challenge: IPF is not an exception to this rule. Different data collection approaches have been applied in measuring the incidence and prevalence of IPF, and the findings of these studies vary widely [1]. A recent study using a sensitive algorithm in the United States found that the incidence and prevalence of IPF, corrected for positive predictive value, were 14.6 per 100,000 person-years and 58.7 per 100,000 persons, respectively [2]. These estimates indicate that, in a large, populated area like the BRIC region, there may be approximately 2 million persons living with IPF. This poses unique challenges to healthcare systems, particularly in an era when effective and safe drugs for IPF are finally available [3]. Thus, the exploration, using first-hand experience, of the challenges and opportunities related to the diagnosis and management of patients with IPF in BRIC countries is of particular relevance. Further, issues such as the availability of high-resolution computed tomography (HRCT) and spirometry or the existence of a multidisciplinary diagnostic environment, which would not present a problem in high-income countries and yet are crucially relevant to the BRIC country context, must be addressed. On the other hand, the rapidly increasing demand for the enrollment of IPF patients in clinical trials of new drugs could lead to BRIC countries becoming a major source of trial participants. This opportunity has been previously explored in a recent phase III trial [4]. In order to ensure that IPF patients obtain an accurate and prompt diagnosis and appropriate access to treatment, field experts, healthcare agencies, and funding bodies must join forces in order to identify sensible and feasible solutions.

### Competing interests

Luca Richeldi was a consultant for and participated in advisory committees of AstraZeneca, Boehringer Ingelheim, GlaxoSmithKline, Promedior, Roche Genentech, Sanofi-Aventis, and UCB. Further, he was a speaker for Boehringer Ingelheim, Cipla Pharmaceuticals, and InterMune. He received research support paid to his institution from InterMune.

## IPF in Brazil

**Fig. 2:**
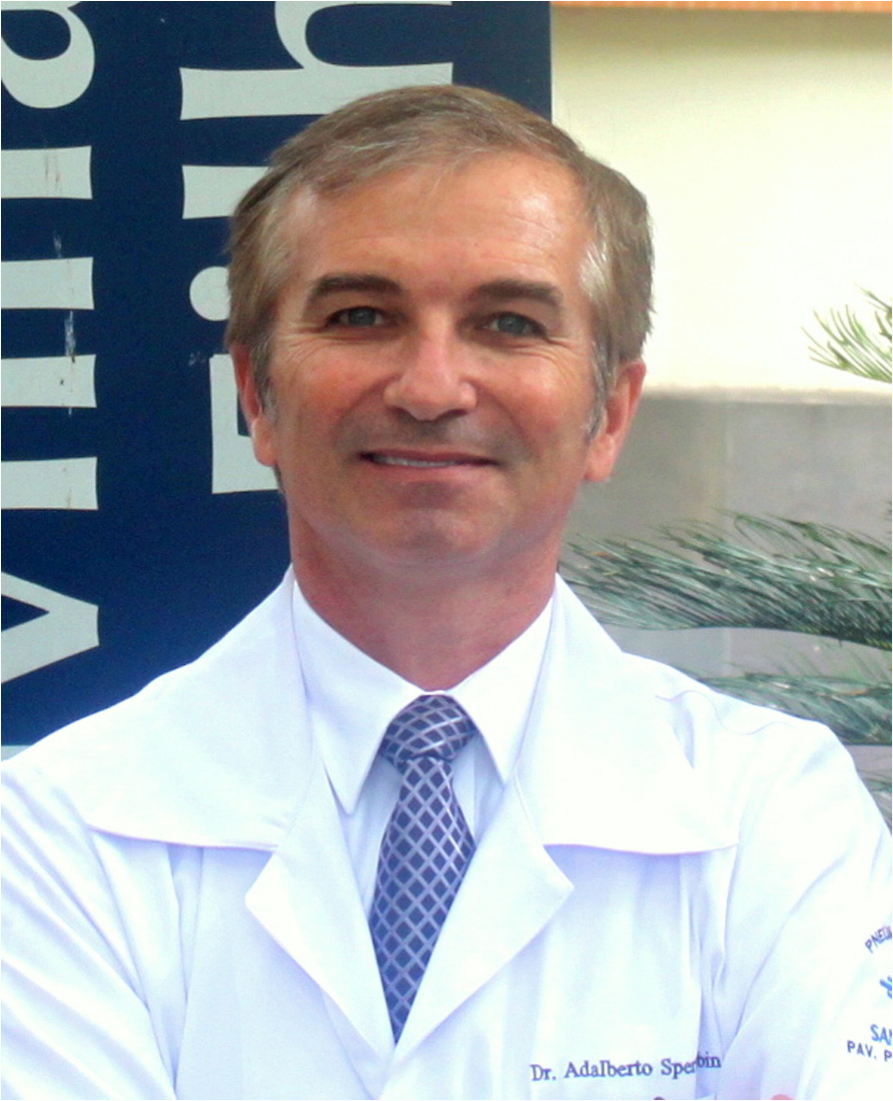
Adalberto Sperb Rubin is Professor of Pulmonary Medicine at the Federal University of Health Sciences of Porto Alegre. He has a PhD from the Federal University of Rio Grande do Sul. He is Chief of the Pulmonary Department of Santa Casa of Porto Alegre and Coordinator of the ILD Center of this hospital. He is currently a member of the American Thoracic Society, the European Respiratory Society, the Latin American Thoracic Association, and the Brazilian Society of Pneumology and Tisiology (SBPT), as well as of the Interstitial Lung Disease Committee of SBPT

Adalberto Sperb Rubin (Fig. 2)

IPF is the most commonly diagnosed ILD in Brazil. Despite epidemiological studies on the Brazilian population being rare [5], IPF has received special attention from the Brazilian pulmonology community [6]. The Brazilian Society of Pneumology and Tisiology (Sociedade Brasileira de Pneumologia e Tisiologia, SBPT) has a very active Interstitial Lung Diseases Committee, which organizes continuing education programs at national symposia and congresses. Brazil has six tertiary referral centers to treat ILDs, and more are being created. In recent years, Brazil has participated in multicenter studies developing new treatments for IPF. Many of the patients involved are still participating in the open phase of these studies, contributing to a better understanding of the long-term effects of these new drugs. Substantial changes to the diagnosis and treatment of IPF, both in Brazil and worldwide, have been recently implemented. These changes have led to easier diagnosis, making histologic biopsies less common; on the other hand, studies have demonstrated that current treatments do not have the anticipated clinical effectiveness [7].

In Brazil, as in many countries, the initial challenge of IPF is based on its proper diagnosis, since this process has significant therapeutic implications. Therefore, IPF, which has as a histologic substratum of usual interstitial pneumonia, must be distinguished from other chronic fibrosing lung diseases as well as from other ILDs. Indeed, in Brazil, cases of chronic hypersensitivity pneumonitis with characteristics very similar to IPF have been recorded [8]. Therefore, whenever possible, the diagnostic process should involve a multidisciplinary group, including a pulmonologist, radiologist, and pathologist (when necessary), with experience in ILDs.

For a number of years, we have followed various international directive agreements and implemented an anti-inflammatory treatment strategy as part of the management of IPF. Recent studies performed in a Brazilian population indicated the possibility that a combination of corticoids and immunosuppressant drugs had beneficial effects [9]. The PANTHER study [3, 7] tested the “triple therapy” (steroids, azathioprine, and N-acetyl cysteine), revealing opposite results to those previously anticipated. As a result of this evidence, the use of this therapeutic strategy is contraindicated, and Brazil’s tertiary referral centers no longer use it.

The relationship between gastroesophageal reflux disease and IPF has long been under evaluation in Brazil [10], as in other countries. Studies have shown that patients receiving anti-reflux treatment, especially proton-pump inhibitors, may have a better survival. Therefore, many patients in Brazil are treated in an empirical way with this medication in order to reduce disease progression. Nevertheless, despite this course of conduct being widely used in current practice, better scientific proof of its effectiveness is required.

As with patients with other chronic lung diseases, IPF patients are included in pulmonary rehabilitation programs, and studies have demonstrated an improvement in various outcomes associated with their quality of life, with the 6-minute walk test used as one of the markers of this benefit [11].

Lung transplantation, recommended in cases where the symptoms are progressive and where functional loss indicates reduced survival, is one of the few therapeutic strategies that has a real impact on survival with IPF [12]. However, this option is limited, especially in Brazil, where few medical centers can perform a procedure of such high complexity and given the low number of organ donors. There are currently two very active centers with established lung transplantation programs in Porto Alegre and São Paulo. The Santa Casa of Porto Alegre Hospital has already performed more than 500 lung transplantations and, currently, more than 50 % of new transplantation cases are IPF patients.

In 2014, two publications in the *New England Journal of Medicine* [13, 14] presented data from multicenter studies on IPF treatment with nintedanib and pirfenidone. Following FDA approval for these drugs, Anvisa – the Brazilian regulatory body – began the process of releasing them in Brazil, with these new treatments predicted to become available in the second half of 2015. These drugs will have a profound impact on the management of IPF in Brazil, since no effective therapy is currently available to treat this population. In 2012, the SBPT published national directives for managing ILDs [5]; it has since become clear that the available therapies have shown little or no clinical evidence of effectiveness.

As is the case in all BRIC countries, the greatest challenges lie in the availability of computed tomography (CT) and the equipment necessary to test lung function in the less developed parts of the country. Further, Brazil lacks a large enough number of pathologists and radiologists with experience in the precise diagnosis of ILDs, something which is fundamental to managing IPF. Nevertheless, there has been rising interest for IPF and its appropriate management within the national medical community, which, along with the availability of new therapies and further exchange with other countries, should lead to an improvement in IPF patient care in Brazil.

### Competing interests

ASB is on the advisory board and a speaker for Boehringer Ingelheim. He has also received funding for clinical research from Boehringer Ingelheim and Roche.

## IPF in Russia

**Fig. 3:**
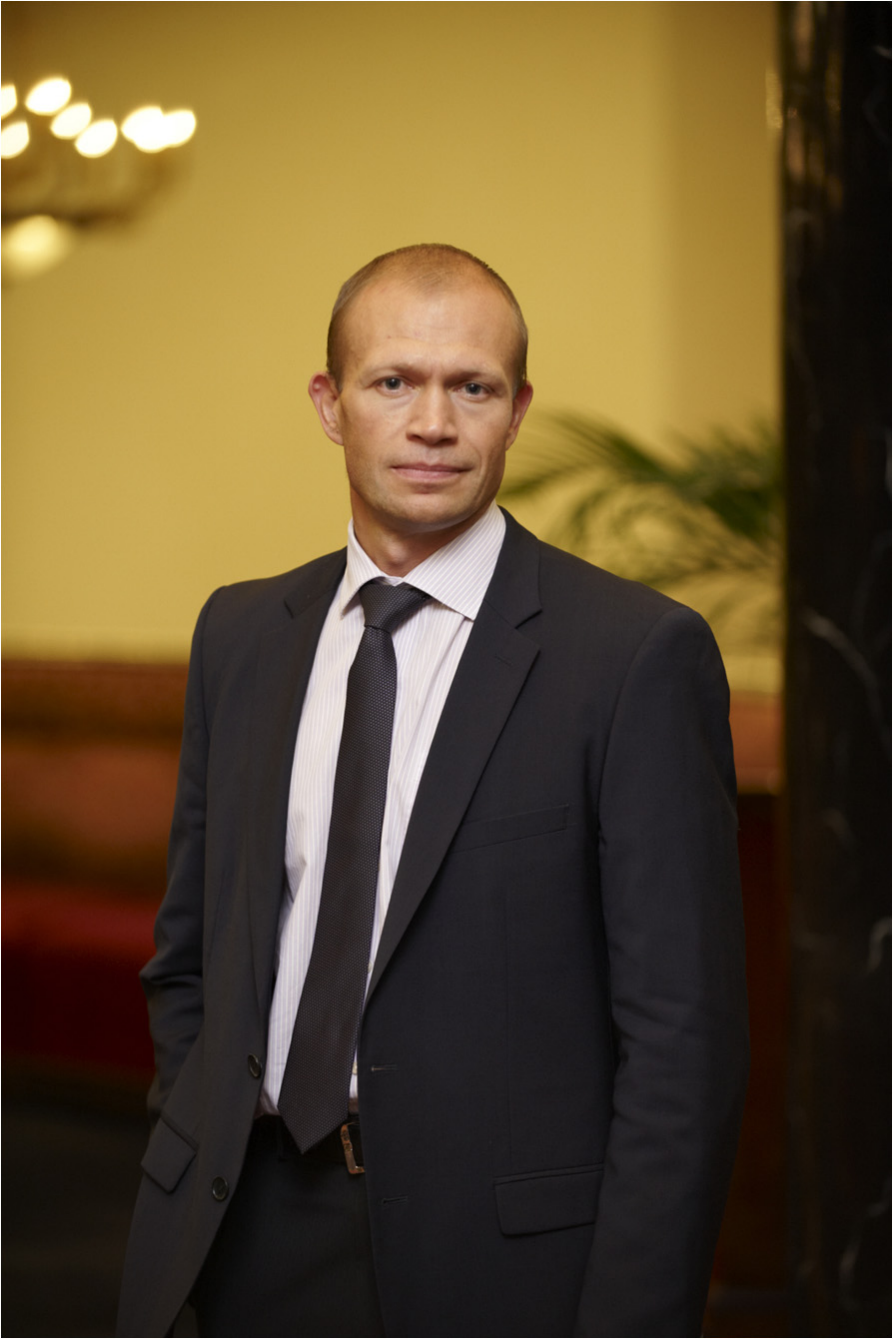
Sergey Avdeev is Professor of Respiratory Medicine at the Pulmonology Research Institute, Moscow. Since 2005, he has held positions as Chair of the Clinical Department and Vice Director of the Institute. His work focuses on the management of advanced pulmonary diseases, diffuse interstitial and rare lung disorders, and pulmonary hypertension. He is also involved in the lung transplant program of Moscow

Sergey Avdeev (Fig. 3)

IPF is currently considered by Russian physicians as a disease that is difficult to diagnose and particularly difficult to treat [15]. To date, there are no national guidelines for the diagnosis and management of IPF in Russia and there is no unique name for the disease – along with the term “IPF” [16], the term “idiopathic fibrosing alveolitis” is also widely used [17]. The majority of IPF patients are followed up by pulmonologists in large multidisciplinary hospitals and outpatient centers in large cities of Russia. Further, the diagnosis of IPF has improved over the past 10 years due to easier accessibility to CT and to the increased awareness of the disease [18].

Epidemiological studies on the prevalence and incidence of IPF in Russia have not been conducted and therefore only rough estimates based on a survey conducted in Moscow are available [19]. Extrapolation of data from this survey suggests that the prevalence and incidence of IPF are approximately 9–11 and 4–6 cases per 100,000 population, respectively. The median estimated survival of IPF patients from the time of diagnosis is only 2–3 years. In order to obtain a clearer picture of the state of IPF in Russia to date, data obtained from a recently conducted national survey of more than 30 leading expert pulmonologists involved in the management of IPF patients [20] is discussed below.

**Fig. 4:**
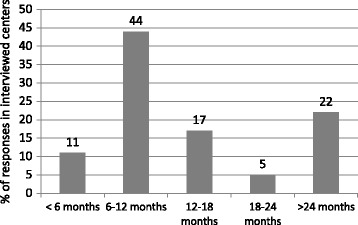
The mean duration of symptoms in idiopathic pulmonary fibrosis patients at the time of initial diagnosis

The data indicates that the average number of IPF patients attending a given participating center over 1 year was 49 (range, 10–150 patients). The participating centers were large multispecialty hospitals (attended 38 % of cases), outpatient centers (28 % of cases), and specialized pulmonology centers (50 % of cases). According to interviewed experts, the average age of patients was more than 60 years (67 % of responses). IPF patients in Russia are characterized by male predominance (66 %) with a high prevalence of smokers (61 %). At the time of initial diagnosis of IPF, the average level of forced vital capacity was 50–80 % of predicted values in 78 % of patients and less than 50 % of predicted values in 22 % of patients. The usual duration of symptoms before the diagnosis of IPF was 6–12 months (44 % of responses), 12–18 months (17 % of responses), or more than 24 months (22 % of responses; Fig. 4). Most often, patients with suspected IPF were sent to participating centers by general physicians (56 %), pulmonologists (22 %), and radiologists (22 %). A surgical lung biopsy for confirmation of a diagnosis of IPF was performed, on average, in 20 % of patients (mainly in young patients and in patients with atypical CT imaging). According to the experts’ answers, 89 % of the interviewed experts had the ability to discuss the results of CT scans with radiologists, 89 % of interviewed pulmonologists had the ability to discuss the results of lung biopsies with morphologists, and 77 % of participating centers had the possibility of facilitating a multidisciplinary discussion about IPF cases.

The primary complaints of IPF patients at the time of initial diagnosis were dyspnea (100 %), cough (56 %), weight loss (11 %), and general weakness (5 %). The initial findings that most frequently revealed IPF were incidental findings on chest X-ray or chest CT (78 %), dyspnea (56 %), “velcro” crackles at lung auscultation (39 %), and cough (28 %). According to the interviewed experts, the most frequent comorbidities and complications in IPF patients were gastroesophageal reflux disease (57 %), cardiovascular disease (54 %), pulmonary hypertension (38 %), emphysema (33 %), sleep apnea (14 %), and lung cancer (3 %).

**Fig. 5:**
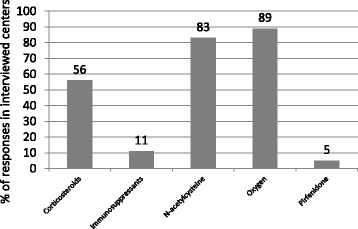
The proportion of physicians who prescribed each of the specified treatments in patients diagnosed with idiopathic pulmonary fibrosis

More than 90 % of IPF patients in Russia receive pharmacological therapy. The most commonly initiated therapies are oral corticosteroids (56 %), immunosuppressants (azathioprine or cyclophosphamide; 11 %), N-acetylcysteine (83 %), long-term oxygen therapy (89 %), anti-reflux agents (72 %), and pirfenidone (5 %; Fig. 5). If oral corticosteroids are prescribed, doses in the majority of centers are low (10–20 mg of prednisolone daily). Pirfenidone and nintedanib are not yet registered in Russia, and therefore their administration is possible only in patients who have the opportunity to obtain the drugs abroad.

A lung transplantation program is being actively developed in Russia [21]. Patients who have successfully undergone a lung transplantation include patients with advanced IPF. However, the main challenges of lung transplantation for IPF patients, as in many other countries, are the shortage of donor organs and the relatively small number of lung transplant centers.

Finally, most interviewed experts (89 %) reported that they used, for the management of IPF patients, the joint recommendations of the American Thoracic Society, the European Respiratory Society, the Japanese Respiratory Society, and the Latin American Thoracic Association (2011), although some also stated that they rely on other recommendations.

### Competing interests

The author declares that he has no competing interests.

## IPF in India

**Fig. 6:**
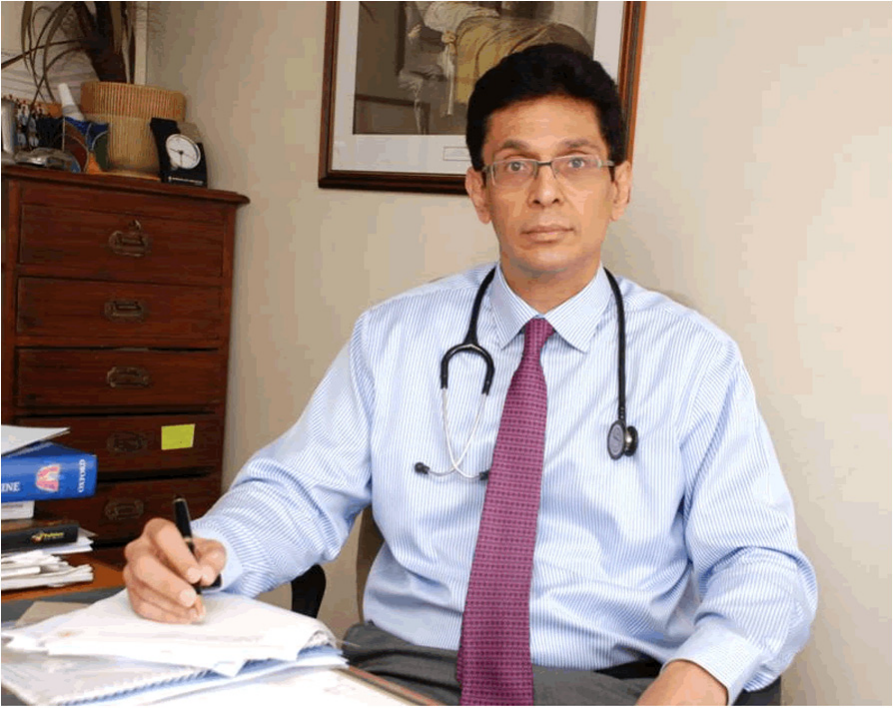
Zarir F. Udwadia is a consultant pulmonologist at Hinduja Hospital and Research Centre, Mumbai, India. His research interests include interstitial lung disease and drug-resistant tuberculosis. He has written over 100 peer-reviewed articles and authored chapters in several international textbooks

Zarir F. Udwadia (Fig. 6)

Most pulmonologists in India will admit to seeing a large number of patients with IPF in their practice and concede that the number seems to be increasing over the years. Nevertheless, this statement cannot be backed up by objective evidence in the form of epidemiological studies of incidence or prevalence since none are available. This section will discuss what is known about IPF in the context of a country of 1.2 billion people and focus on its distinctive features in the Indian setting.

There has been a recent surge in the recognition of IPF in India. The initial Indian report of IPF occurred in 1979 by Jindal et al. [22], from the Postgraduate Institute of Medical Education and Research, the country’s most esteemed post-graduate medical institution in Chandigarh. The study reported that almost 46 % of patients (n = 61) with diffuse parenchymal lung disease (DPLD) had IPF. A few years later, Mahashur et al. [23], from another large teaching hospital in Bombay, reported that IPF accounted for 50 % of cases in a mixed group of 63 patients with DPLDs; the advantage of this study was that open lung biopsies were performed in the majority of patients. In 1984, a larger study by Sharma et al. [24], from New Delhi, showed that IPF accounted for 28 % of patients with ILD. Since then, there have been isolated studies by Maheshwari et al. [25] and Subhash et al. [26] in 2004, Sen and Udwadia in 2010 [27], and Kundu et al. in 2014 [28], from centers representing all the major regions in the country, all demonstrating that IPF accounted for significant numbers of patients with DPLDs in India. The major findings from these studies are summarized in Table 1.

**Table 1:** Studies reporting idiopathic pulmonary fibrosis (IPF) in India

Study authors [ref.]	Study population	Proportion with IPF/main findings
Jindal et al. [22]	61 with diffuse parenchymal lung disease (DPLD), 5 years	46 %
Sharma et al. [24]	133 patients with DPLD	28.6 %
Maheshwari et al. [25]	76 patients with IPF	Female preponderance
Subhash et al. [26]	97 patients with DPLD	43 %
Sen and Udwadia [27]	274 patients with DPLD, 7 years	43 %
Kundu et al. [28]	92 patients	38 %

Thus, available epidemiological data is limited to a few studies, all of which were retrospective. Nevertheless, these studies focused on different parts of India and showed no regional variations, and they all indicated that IPF is the most common cause of DPLD, being more common than other DPLDs such as sarcoidosis, hypersensitivity pneumonia, and ILD secondary to collagen vascular disease.

Unfortunately, these studies do not provide information on the prevalence of IPF. If one extrapolates even the more conservative prevalence of 10 cases per 100,000 from Gribbin’s IPF data from the UK [29], this translates into at least 130,000 potential IPF patients in India. These numbers, coupled with physicians’ empirical observations of numbers being seemingly on the rise, indicate that IPF will pose a significant disease burden. Whether this is a true increase, or represents an aging Indian population or increased physician awareness coupled with improved availability and quality of CT scanning, is difficult to determine. Nevertheless, an important clinical observation is that IPF in India is generally diagnosed at an advanced stage due to various reasons, as discussed below.

Overlap with tuberculosis (TB):TB still dominates respiratory medicine in India and there is a tendency to initially mislabel patients with IPF as TB. These patients are subjected to long and unfruitful anti-TB drug trials before the correct diagnosis is finally considered [30]. Whilst the infiltrates produced by TB can rarely be mistaken for fibrosis, and despite the fact that as many as 65 % of patients with TB are left with residual fibrosis at the end of their treatment (Udwadia ZF, Shah H, unpublished data), mislabeling rarer DPLDs as TB requires a change in mindset which will materialize with better education of general physicians and internists.

Over reliance on chest radiography:In a resource-limited setting like India, a chest radiograph is always the initial, and sometimes only, diagnostic test a patient from the rural interiors undergoes. Whilst chest radiography has a low sensitivity at the best of times, the quality of some of these radiographs is so poor that IPF is often never suspected and further work up never initiated.

Poor HRCT penetrance: The first attempts at HRCT of the lungs began in centers in Mumbai and Delhi in 1991. The currently installed CT scanner base of 5,000 machines in the country is woefully inadequate to meet the needs of the population. India’s CT penetrance of one scanner per million population is much less than that of most of its BRIC neighbors. In addition, the majority of scanners (outside of the country’s main metropolitan areas) are of poor quality and incapable of imaging at 1-mm slice thickness. Countrywide, there are only a few radiographers with a special interest in chest imaging who will provide good quality images and their interpretation.

Pulmonary function test unavailability:In smaller rural centers, though spirometry is widely performed, diffusion capacity is seldom available and the 6-minute walk testing rarely performed.

Surgical biopsies and their interpretation: Although surgical biopsies are currently less commonly required in IPF since HRCT scanning has assumed a pivotal position in the diagnostic algorithm, on the occasions in which they do need to be performed, there are very few surgeons across the country capable of performing video-assisted surgical biopsies and even fewer lung pathologists capable of interpreting them accurately.

Treatment of IPF in India has followed global trends: initial treatment revolved around high dose steroids and was followed by triple therapy with steroids, azathioprine, and N-acetyl cysteine; current treatment revolves around the use of pirfenidone. Interestingly, this novel anti-fibrotic drug has been available in generic form through two Indian pharmaceutical companies since 2010, several years before it received European Union and National Institutes of Health approval. Steroids are still used freely and in large doses, despite a large body of evidence suggesting they do more harm than good. Steroids, if used at all in IPF, must be used with great caution in the Indian context – the world’s largest diabetic (65 million) and TB populations (2.2 million cases) reside in this country and the potential for harm to these patients is great. India also has a huge population of patients with latent TB infection and the risk of activation of TB in these patients when given steroids for IPF is high.

Lung transplantation for IPF has yet to make a foothold in this country. The first lung transplantation for IPF was performed at the Hinduja Hospital as recently as 2012. This patient survived only 3 months and the handful of cases subsequently transplanted from a few other centers across the country have also met limited success.

IPF in India is widely prevalent but countrywide epidemiological studies are needed to map its exact incidence and prevalence. It is often misdiagnosed or diagnosed late (the previously described “TB effect”) and is often treated with traditional immunosuppression despite the more widespread availability of pirfenidone. Thus, centers of excellence where expert pulmonologists, radiologists, and pathologists can see these patients in consensus are required. Lung transplantation must also be expanded with the major hospitals in the larger cities offering this much needed option to IPF patients. A nationwide electronic ILD registry has recently been established and has already enrolled 700 patients from at least 20 different centers across the country [31]; this will provide important data and an evidence base for future research into IPF in India.

### Competing interests

The author declares that he has no competing interests.

## IPF in China

Zuo Jun Xu (Fig. 7)

**Fig. 7:**
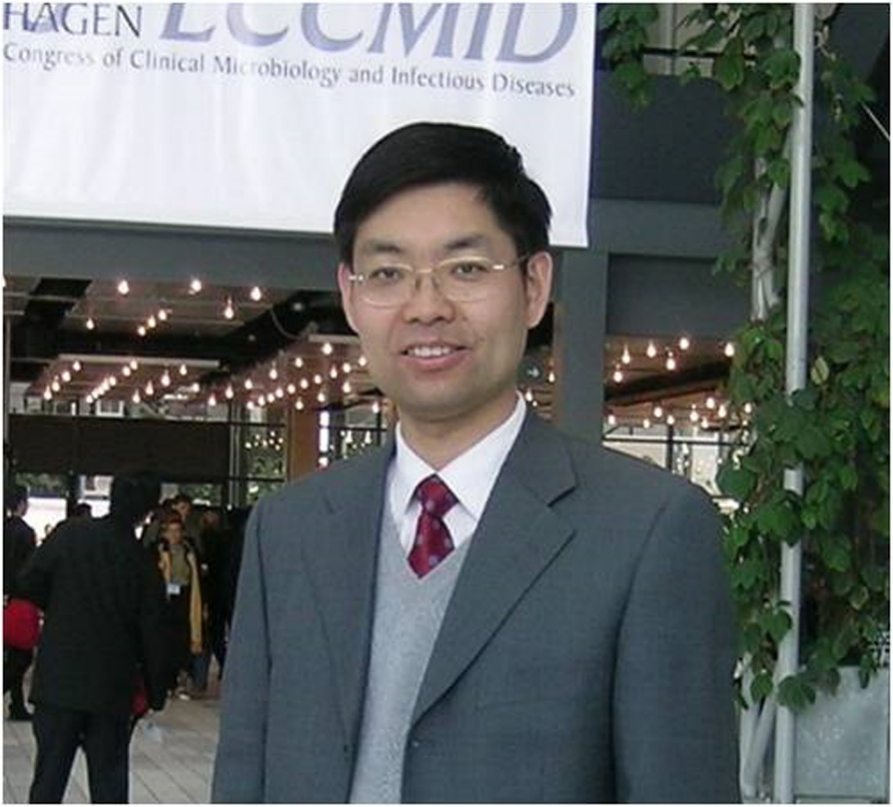
Zuo Jun Xu, is the Chief Physician and a Professor at the Respiratory Disease Department of Peking Union Medical College Hospital, Peking Union Medical College and Chinese Academy of Medical Sciences, and is also the Vice Director of the Interstitial Lung Disease Group of the Chinese Society of Respiratory Diseases, Chinese Medical Association. He is a member of the Council of Beijing Medical Doctor Association as well as a member of the Standing Committee of the Beijing Association of Traditional Chinese Medicine and Western Medicine. He has published over 200 scientific papers and participated in the editing of 65 monographs

ILD, also referred to as DPLD, is a heterogeneous group of diseases that mostly involves the pulmonary interstitium. IPF is a specific form of chronic, progressive fibrosing idiopathic interstitial pneumonia [32]. To date, there have been no nationwide or large-scale prospective epidemiological studies of the clinical treatment of different types of ILD (including IPF) patients in China. However, a number of medical centers focus on the diagnosis and treatment of ILD as a respiratory disease specialty and follow international standards regarding the diagnostic procedures and treatment of IPF patients. In China, the diagnosis and treatment of IPF, as well as related research, are generally conducted in hospitals affiliated with medical schools, namely teaching hospitals. The highest official guidance institution for ILD in China is the Interstitial Lung Disease Study Group of the Chinese Thoracic Society, which is responsible for the introduction of the latest international consensus and research progress on ILD to clinicians specialized in respiratory medicine throughout the country via lectures and articles, as well as for nationwide clinical research related to ILD. The classification and naming of idiopathic interstitial pneumonias in China strictly follow the relevant international guidelines.

To date, China has not conducted any national population-based epidemiological surveys on IPF and, therefore, no epidemiological data of IPF in the Chinese population are currently available. Only a few analyses have been conducted on the diagnosis and treatment data of hospitalized ILD patients in several local areas [33–36]. A 2003 study from the Interstitial Lung Disease Group of the Chinese Thoracic Society revealed that the 10 hospitals surveyed admitted and treated 56 ILD cases in 1990, accounting for 1.98 % of the yearly hospitalized cases in the Department of Respiratory Medicine, while the number rose to 485 ILD cases in 2003, accounting for 4.66 % of the yearly hospitalized cases [33]. A single-center study by Li et al. [34] found that a total of 395 ILD patients were admitted from 1990 to 2003; of these, 58 ILD patients (14.7 %) were admitted from 1990 to 1997 and 395 ILD patients (85.3 %) from 1998 to 2003. The 2008 data from the Chongqing Interstitial Disease Collaborative Group showed that ILD cases admitted to the five surveyed teaching hospitals in 2002 accounted for 2.8 % of all hospitalized cases in the Department of Respiratory Diseases in that year, with the ratio increasing to 8.3 % in 2006 [35]. A 2012 study by Wei et al. [36] including 10 hospitals in Tianjin, suggested that hospitalized ILD cases accounted for 4.5 % of all hospitalized cases in the Department of Respiratory Diseases in 2003, while this ratio increased to 39.5 % in 2009. The above data revealed that, after accounting for the changes in the Chinese population [6], the incidence of ILD in China showed a gradual upward trend after 2000. This upward trend may be related to improvement in relevant ILD diagnostic techniques and increased ILD awareness [32], as well as a possible real increase in ILD incidence [33].

Two of the studies mentioned above, including 3,568 patients with ILD, found that idiopathic interstitial pneumonias were seen in 53 % of patients (1,891 cases), IPF in 25.1 % (894 cases), connective tissue disease-associated ILD in 14 % (499 cases), sarcoidosis in 4 % (144 cases), and extrinsic allergic alveolitis in 3.8 % (100 cases) [33, 36]. A retrospective study by Zhang et al. [37] analyzed 418 patients with ILD diagnosed by lung biopsy from 1999 to 2009 in China and showed that idiopathic interstitial pneumonias were found in 41.9 % of patients. Of these, 35.4 % (62 cases) were found to be IPF, followed by unclassifiable interstitial pneumonia (21.7 %; 38 cases), non-specific interstitial pneumonia (19.4 %; 34 cases), and chronic obstructive pulmonary disease (13.7 %; 24 cases).

The various hospital categories exhibit substantial differences in the diagnosis of IPF. At most community- or county-level hospitals, IPF is often missed or misdiagnosed. Many IPF patients are often first mistakenly diagnosed with pneumonia or chronic obstructive pulmonary disease. In addition, due to insufficient awareness of ILD, physicians sometimes misdiagnose alveolar protein deposition disease, certain secondary ILDs, and certain infectious pneumonias as idiopathic interstitial pneumonitis and treat these diseases with corticosteroids with/without immunosuppressive therapy. However, in hospitals that specialize in the diagnosis and treatment of ILD, the procedures for IPF diagnosis and treatment are highly standardized. For patients with pathological data, the diagnostic guideline is to establish a final diagnosis based on discussion among clinical experts, radiologists, and pathologists who are specialized in ILD. In hospitals that do not have the capacity for C-reactive protein diagnosis, physicians sometimes transfer the related data to teaching hospitals with the appropriate capabilities for consultation.

Fourteen sites in China participated in the recent phase III international clinical study using nintedanib for the treatment of IPF [14]. A total of 153 patients with ILD were screened, 101 patients were randomly enrolled, and the overall diagnostic accuracy rate was 66 %. Among the 25 patients with ILD screened at our center, 24 patients showed HRCT images meeting the IPF diagnostic criteria, with a diagnostic accuracy rate of 96 %. The different diagnostic accuracy rates reflect the gaps in diagnostic capacities among various hospitals in China and between China and the international medical field.

In China, several problems exist in the diagnosis and treatment process of IPF patients. Firstly, most hospitals have chest radiologists, but only a few have radiologists specialized in ILD. The lack of specialized radiologists, to some extent, results in respiratory disease specialists having a better understanding of ILD chest imaging than radiologists, thus weakening the role of radiologists in the diagnosis of IPF. In addition, chest HRCT examination is not universal, and many basic primary hospitals are not able to perform these, which is one of the reasons that many primary hospitals cannot determine a correct diagnosis of IPF. Second, although some hospitals have pathologists with expertise in ILD, the vast majority of hospital pathologists in China are general pathologists, and there are almost no specialized lung pathologists in the entire country. This problem may also be shared by most other hospitals outside China. Finally, because the Chinese healthcare system does not have an established referral system, most hospitals still do not have the capacity to systematically manage the relevant clinical data of outpatients, and the information of IPF patients who only receive outpatient treatment cannot be systematically organized/managed.

The majority of ILD treatment centers in China are able to conform to international standards in terms of IPF treatment regimens by studying the related literature, updating treatment programs for IPF patients, and standardizing the treatment of these patients. Further, many new drugs can enter the Chinese market in a timely manner given the increased opening of trade. With the improvement of basic pharmaceutical research capacities, China also has several new IPF drugs that were originally developed domestically, such as pirfenidone. In addition, traditional Chinese medicine and therapy are unique to China, and attempts to treat IPF using these traditional methods, including certain results from basic and clinical research, suggest that some Chinese medicines may improve the symptoms and prognosis of the disease [38–42]. Finally, through the introduction and further improvement of organ transplantation in China, as well as national benefits for the medical costs of organ transplant patients, there have been recent reports of lung transplantations in ILD patients [43].

However, there are still some shortcomings in IPF patient care. First, due to deficiencies in the follow-up of patients in the Chinese healthcare system, the vast majority of hospitals do not conduct systematic follow-up of ILD patients. Many IPF patients seek treatment at multiple hospitals, and therefore their information is used repeatedly. Second, limited by the realities of new drug approval in China, many new drugs will take 3–5 years longer to enter the market compared to those in other countries. Third, although basic research results have suggested that traditional Chinese medicine can improve pulmonary fibrosis and has been widely used for the treatment of IPF in China, the specific mechanism of action and the identities of functional components in traditional Chinese medicine remain unclear. In addition, traditional Chinese medicine treatment requires individualization, and it is very difficult to apply a single prescription to all patients or to conduct double-blind studies. Therefore, there have been no large randomized controlled trials on IPF treatment using traditional Chinese medicine. Finally, due to the extreme lack of lung donors and the high cost of organ transplantation, as well as traditional resistance to organ transplantation in China, only a few IPF patients receive lung transplants each year.

In summary, ILD incidence has shown a gradual increase in China. Teaching hospitals with ILD treatment centers or specialized in ILDs are able to keep pace with the development of international IPF treatments and standardize the treatment of IPF patients. However, given the condition of China’s healthcare system, further improvements are required for IPF diagnosis and treatment.

### Competing interests

The author declares that he has no competing interests.

## References

[1] Kaunisto J, Salomaa ER, Hodgson U, Kaarteenaho R, Myllarniemi M (2013). Idiopathic pulmonary fibrosis--a systematic review on methodology for the collection of epidemiological data. BMC Pulm Med.

[2] Esposito DB, Lanes S, Donneyong M, Holick CN, Lasky JA, Lederer D, et al. Idiopathic pulmonary fibrosis in US automated claims: incidence, prevalence and algorithm validation. Am J Respir Crit Care Med. 2015. Ahead of print. 10.1164/rccm.201504-0818OC 26241562

[3] Karimi-Shah BA, Chowdhury BA (2015). Forced vital capacity in idiopathic pulmonary fibrosis--FDA review of pirfenidone and nintedanib. N Engl J Med.

[4] Richeldi L, Costabel U, Selman M, Kim DS, Hansell DM, Nicholson AG (2011). Efficacy of a tyrosine kinase inhibitor in idiopathic pulmonary fibrosis. N Engl J Med.

[5] Fortuna FP, Perin C, Cunha L, Rubin AS (2003). Mortality due to idiopathic pulmonary fibrosis in the State of Rio Grande do Sul (Brazil). J Bras Pneumol.

[6] Baldi BG, Pereira CA, Rubin AS, Santana AN, Costa AN, Carvalho CR (2012). Highlights of the Brazilian Thoracic Association guidelines for interstitial lung diseases. J Bras Pneumol.

[7] Raghu G, Anstrom KJ, King TE, Lasky JA, Martinez FJ, Idiopathic Pulmonary Fibrosis Clinical Research Network (2012). Prednisone, azathioprine, and N-acetylcysteine for pulmonary fibrosis. N Engl J Med.

[8] Lima MS, Coletta EN, Ferreira RG, Jasinowodolinski D, Arakaki JS, Rodrigues SC (2009). Subacute and chronic hypersensitivity pneumonitis: histopathological patterns and survival. Respir Med.

[9] Pereira CA, Malheiros T, Coletta EM, Ferreira RG, Rubin AS, Otta JS (2006). Survival in idiopathic pulmonary fibrosis-cytotoxic agents compared to corticosteroids. Respir Med.

[10] Bandeira CD, Rubin AS, Cardoso PF, Moreira Jda S, Machado Mda M (2009). Prevalence of gastroesophageal reflux disease in patients with idiopathic pulmonary fibrosis. J Bras Pneumol.

[11] Florian J, Rubin A, Mattiello R, Fontoura FF, Camargo Jde J, Teixeira PJ (2013). Impact of pulmonary rehabilitation on quality of life and functional capacity in patients on waiting lists for lung transplantation. J Bras Pneumol.

[12] Machuca TN, Schio SM, Camargo SM, Lobato V, Costa CD, Felicetti JC (2011). Prognostic factors in lung transplantation: the Santa Casa de Porto Alegre experience. Transplantation.

[13] King TE, Bradford WZ, Castro-Bernardini S, Fagan EA, Glaspole I, Glassberg MK, ASCEND Study Group (2014). A phase 3 trial of pirfenidone in patients with idiopathic pulmonary fibrosis. N Engl J Med.

[14] Richeldi L, du Bois RM, Raghu G, Azuma A, Brown KK, Costabel U (2014). INPULSIS Trial Investigators. Efficacy and safety of nintedanib in idiopathic pulmonary fibrosis. N Engl J Med.

[15] Petrov DV, Ovsjannikov NV, Kapralov EA, Kapustian OV (2014). Interstitial lung diseases: point of view of practical doctor. Practical Pulmonol.

[16] Chuchalin AG (2000). Idiopathic pulmonary fibrosis. Ther Archive.

[17] Ilkovich ММ, Novikova LN, Koroleva MG (2003). Idiopathic fibrosing alveolitis: controversies in modern presentations. Pulmonology.

[18] Kotlyarov PM, Georgiadi SG (2005). New technologies and progress of imaging of diffuse interstitial lung diseases. Pulmonology.

[19] Avdeev SN (2014). Idiopathic pulmonary fibrosis: current concepts and diagnostic approaches. Practical Pulmonology.

[20] Avdeev SN. Idiopathic pulmonary fibrosis: approaches to the diagnosis and treatment in Russia. Pulmonology. 2015. Ahead of print. In Russian.

[21] Khubutia MS, Chuchalin AG, Abakumov MM, Avdeev SN, Timerbaev VK, Poplavsky IV (2011). The first lung transplantation at the Research Institute of Emergency named after N.V. Sklifosovsky. Transplantation.

[22] Jindal SK, Malik SK, Deodhar SD, Sharma BK (1979). Fibrosing alveolitis: a report of 61 cases seen over the past five years. Indian J Chest Dis Allied Sci.

[23] Mahashur AA, Dave KM, Kinare SG, Kamat SR, Shetye VM, Kolhatkar VP (1983). Diffuse fibrosing alveolitis – an Indian experience. Lung India.

[24] Sharma SK, Pande JN, Verma K, Guleria JS (1989). Bronchoalveolar lavage fluid (BALF) analysis in interstitial lung diseases--a 7-year experience. Indian J Chest Dis Allied Sci.

[25] Maheshwari U, Gupta D, Aggarwal AN, Jindal SK (2004). Spectrum and diagnosis of idiopathic pulmonary fibrosis. Indian J Chest Dis Allied Sci.

[26] Subhash HS, Ashwin I, Solomon SK, David T, Cherian AM, Thomas K (2004). A comparative study on idiopathic pulmonary fibrosis and secondary diffuse parenchymal lung disease. Indian J Med Sci.

[27] Sen T, Udwadia ZF (2010). Retrospective study of interstitial lung disease in a tertiary care centre in India. Indian J Chest Dis Allied Sci.

[28] Kundu S, Mitra S, Ganguly J, Mukherjee S, Ray S, Mitra R (2014). Spectrum of diffuse parenchymal lung diseases with special reference to idiopathic pulmonary fibrosis and connective tissue disease: An eastern India experience. Lung India.

[29] Gribbin J, Hubbard RB, Le Jeune I, Smith CJ, West J, Tata LJ (2006). Incidence and mortality of idiopathic pulmonary fibrosis and sarcoidosis in the UK. Thorax.

[30] Udwadia ZF, Sen T, Jindal SK, du Bois RM, Richeldi L (2009). Interstitial lung diseases in a resource-limited setting: the case of India. European Respiratory Monograph 46: Interstitial Lung Diseases.

[31] Singh V, Sharma BB (2014). Laying the ground for research of interstitial lung disease in our country: ILD India registry. Lung India.

[32] Raghu G, Rochwerg B, Zhang Y, Garcia CA, Azuma A, Behr J (2015). An Official ATS/ERS/JRS/ALAT Clinical Practice Guideline: treatment of idiopathic pulmonary fibrosis. An update of the 2011 Clinical Practice Guideline. Am J Respir Crit Care Med.

[33] Interstitial Lung Disease Group of Chinese Society of Respiratory Disease (2004). Summary of the sixth national symposium on respiratory diseases in China. Zhong hua Jie He He Hu Xi Za Zhi.

[34] Li HP, Huang JA, Fan F, Li X, Yu H, Zhao L (2007). Clinical analysis of 395 patients with diffuse lung diseases. Shanghai Med J.

[35] Yang XM, Wang CZ, Cui SH, Liu Z, Li XJ, Du XZ (2008). Retrospective analysis of diffuse interstitial lung disease cases admitted in 5 teaching hospitals in Chongqing. Chin J Respir Crit Care Med.

[36] Wei LQ, Peng SC, Cao J, Liu GY, Lai YP, Jia W (2012). Clinical features and diagnosis and treatment of diffuse interstitial lung disease: a multi-center study. Chinese General Practice.

[37] Zhang D, Liu Y (2010). Surgical lung biopsies in 418 patients with suspected interstitial lung disease in China. Intern Med.

[38] Ji Y, Wang T, Wei ZF, Lu GX, Jiang SD, Xia YF (2013). Paeoniflorin, the main active constituent of Paeonia lactiflora roots, attenuates bleomycin-induced pulmonary fibrosis in mice by suppressing the synthesis of type I collagen. J Ethnopharmacol.

[39] He H, Tang H, Gao L, Wu Y, Feng Z, Lin H (2015). Tanshinone IIA attenuates bleomycin-induced pulmonary fibrosis in rats. Mol Med Rep.

[40] Li L, Li D, Xu L, Zhao P, Deng Z, Mo X (2015). Total extract of Yupingfeng attenuates bleomycin-induced pulmonary fibrosis in rats. Phytomedicine.

[41] Xu H, Li S (2010). Pharmacological effects of Bailing capsule and its application in lung disease research. Zhongguo Zhong Yao Za Zhi.

[42] Zhang W, Jiang LD, Zhang XM, Wu JJ, Deng Y, Lu XF (2008). Influence of feixian formula on serum transforming growth factor-beta1 and platelet-derived growth factor in rats with bleomycin-induced pulmonary fibrosis. Zhong Xi Yi Jie He Xue Bao.

[43] Mao W, Chen J, Zheng M, Wu B, Zhu Y (2013). Initial experience of lung transplantation at a single center in China. Transplant Proc.

