# Correction to: Idiopathic pulmonary fibrosis in BRIC countries: the cases of Brazil, Russia, India, and China

**DOI:** 10.1186/s12916-021-02111-4

**Published:** 2021-09-05

**Authors:** Luca Richeldi, Adalberto Sperb Rubin, Sergey Avdeev, Zarir F. Udwadia, Zuo Jun Xu

**Affiliations:** 1 Southampton Respiratory Biomedical Research Unit, Mailpoint 813, LE75 E Level, South Academic Block, University Hospital Southampton NHS Foundation Trust, Southampton, SO16 6YD UK; 2 Federal University of Porto Alegre (UFCSPA), Pulmonary Department of Santa Casa Hospital, Porto Alegre, Brazil; 3 Clinical Department, Pulmonology Research Institute, 32, 11-th Parkovaya str., Moscow, 105077 Russia; 4 Hinduja Hospital and Research Centre, Mahim, Mumbai, 400016 India; 5 Breach Candy Hospital, B. Desai Road, Mumbai, 400026 India; 6 Department of Respiratory Medicine, Peking Union Medical College Hospital, Chinese Academy of Medical Sciences & Peking Union Medical College, Beijing, 100730 China

**Correction to: BMC Med 13, 237 (2015)**

**https://doi.org/10.1186/s12916-015-0495-0**

There was missing information in the Competing Interests section of our published article [1].

The statement authored by Zarir F. Udwadia should read as follows:

“ZUF has participated in trials for Cipla Pharmaceutical and Boehringer for Pirfenidone and Nintedanib, two of the drugs that were referred to in the article.”
